# Prognostic value of alcohol consumption and some other dietary habits for survival in a cohort of Chinese men with lung cancer

**DOI:** 10.1186/s40880-017-0188-5

**Published:** 2017-02-10

**Authors:** Wentao Li, Lap Ah Tse, Joseph S. K. Au, Kai Shing Yu, Feng Wang, Ignatius Tak-sun Yu

**Affiliations:** 1 JC School of Public Health and Primary Care, The Chinese University of Hong Kong, 4/F School of Public Health and Primary Care, Prince of Wales Hospital, Sha Tin, N.T., Hong Kong SAR, China; 2 Department of Clinical Oncology, Hong Kong Adventist Hospital, Hong Kong SAR, China

**Keywords:** Dietary habits, Alcohol, Lung cancer, Prognosis, Epidemiology

## Abstract

**Background:**

Alcohol consumption and some other dietary habits are thought to be associated with lung cancer incidence. However, the effects of these habits on lung cancer prognosis have been studied rarely. The purpose of this study was to address these gaps in knowledge.

**Methods:**

We studied a cohort of 1052 Chinese men in Hong Kong who were diagnosed with primary lung cancer. Cox proportional hazards models were used to determine the prognostic values of consumption of alcohol, fresh fruits or vegetables, meat, and fried or preserved food.

**Results:**

Compared with never drinkers, men who drank alcohol 1–3 days per week had a more favorable lung cancer prognosis (hazard ratio [HR]: 0.82, 95% confidence interval [CI] 0.68–0.97); however, this survival advantage was not significant in men who drank alcohol more frequently (HR: 0.91, 95% CI 0.73–1.14). Compared with men who consumed preserved or fried food only occasionally, men who consumed these foods frequently had a higher risk of lung cancer mortality (HR: 1.20, 95% CI 1.00–1.42).

**Conclusions:**

Occasional consumption of alcohol was a favorable survival factor for Chinese men with lung cancer. However, this survival benefit did not exist for frequent drinkers of alcohol. Chinese men with lung cancer who were frequent consumers of fried or preserved food had a worse prognosis than those who consumed these foods only occasionally.

## Background

Worldwide, for centuries lung cancer has been the leading cause of cancer-related death [1]. In China, lung cancer is the most common and most deadly type of cancer [2, 3]. Because of the deterioration of air quality, the incidence of lung cancer is expected to increase [4]. The association between lung cancer risk and lifestyle is an emerging concern. However, the findings of several studies on the association between frequent alcohol drinking and lung cancer risk have been controversial and unconvincing, mainly because of the residual confounding effect of tobacco smoking [5, 6]. Meanwhile, epidemiologic studies have shown that lung cancer risk is inversely related to the frequent consumption of fruits or vegetables [7, 8] and positively associated with meat consumption [9–12]. A previous study of ours indicated that the frequent consumption of preserved or fried food is also related to lung cancer risk [12].

Many studies have focused on the relationship between lifestyle and lung cancer risk; in contrast, very few studies have investigated the association between lifestyle and the prognosis of lung cancer patients. This is perhaps because most of the studies on lung cancer survival have been focused on the prognostic value of tumor characteristics and treatments. However, some either carcinogenic or protective habits may have significant effects on cancer progression; some carcinogens may also have the potential to promote tumor progression. Furthermore, lifestyle habits are modifiable, and cancer patients may improve their prognosis by adopting more favorable habits. To date, no study has investigated the effects of consumption of alcohol, meat, and fried or preserved food on lung cancer prognosis, and very few studies have examined the association of fresh fruit or vegetable consumption with lung cancer prognosis. The purpose of this study was to address these gaps in knowledge.

## Methods

### Study population

We consecutively recruited 1208 Chinese men who had histologically confirmed lung cancer. These patients were from a completed population case–control study conducted at the Department of Clinical Oncology of Queen Elizabeth Hospital in Hong Kong during the period of February 2004 to September 2006. The response rate was 96%. Patients were considered eligible if they were diagnosed with primary lung cancer. Patients who were older than 80 years were excluded. The Department of Clinical Oncology of Queen Elizabeth Hospital, which serves approximately one-fourth of all local cases, is the largest lung cancer center in Hong Kong. The age distribution of patients and the histologic subtypes of lung cancer that were reported by the Queen Elizabeth Hospital were similar to those reported by the Hong Kong Cancer Registry [12]. Details of the recruitment process have been described previously [13, 14]. Ethics agreements of the study were obtained from the ethics committees of both the Chinese University of Hong Kong and Queen Elizabeth Hospital (KC/KE 04–0014/ER–1, KC/KE 08–0028/ER–2). Written consent forms were obtained from all patients.

### Information collection

A structured questionnaire was used by trained interviewers to collect information on patients’ cigarette smoking, alcohol consumption, and dietary habits, as well as other related factors. Patients were classified as never smokers (smoking <20 packs of cigarettes in a lifetime or ≤1 cigarette per day for 1 year); former smokers (quit smoking ≥2 years ago); and current smokers (still smoking or quit <2 years ago). Current and former smokers were asked for information about daily cigarette smoking, years of smoking, and years since cessation (if they quit). In terms of alcohol consumption, patients were asked to report whether they had consumed alcoholic beverages [beer, red wine, white wine (including rice wine), and liquor] during the past year. If the answer was “yes,” they were classified as drinkers, and then they were asked about their frequency of consumption. Patients who drank alcohol 1–3 days per week were classified as occasional drinkers, whereas those who drank more often were classified as frequent drinkers. We did not, however, collect information about the quantity of alcohol (i.e., grams per day) that the patients consumed. In terms of dietary habits, patients were asked for information on their consumption of fresh fruits or vegetables, meat, and preserved or fried food. Patients were classified as occasional consumers if their average consumption was less than one serving per day (one serving = 80 g); if patients consumed one or more servings per day, they were classified as frequent consumers. Information on body mass index (BMI, kg/m^2^), age at diagnosis, comorbidity (translated into the Charlson Comorbidity Index), cancer stage at diagnosis, and treatment type (surgery, chemotherapy, radiotherapy, alternative therapy, or combination therapy) was obtained from patients’ referral letters, medical records, and the clinical management system of Hong Kong. Additionally, information on histologic differentiation was obtained from pathologic reports.

### Follow-up

The vital status of each patient was obtained by a passive surveillance method. This method involved reviewing information from several sources, including clinical discharge notes and the clinical management system of Hong Kong. The follow-up start date was the date of each patient’s pathologic diagnosis of lung cancer. The last follow-up was conducted on December 31, 2008. Patients whose vital status could not be ascertained were considered lost to follow-up. The primary endpoint was all-cause mortality or the last follow-up.

### Statistical analysis

The Mantel–Haenszel Chi square test and Fisher’s exact test were used for distribution analyses. An independent *t* test and an analysis of variance test were used to compare means. Overall survival was considered the prognosis endpoint. After examining the proportionate assumption, Cox proportional hazards models were used to calculate the hazard ratios (HRs) and 95% confidence intervals (CIs). Potential confounders were required to be associated with drinking/dietary habits and the survival outcome. Initially, we included the following as potential confounders in the “base” model: district of residence, age group, education level, marital status, family income, smoking status, smoking pack-years, years after smoking cessation, cancer history in first-degree relatives, incense burning habit, age at diagnosis, BMI, Charlson Comorbidity Index score, cancer stage at diagnosis, and treatment type. Variables that could alter the estimate by 10% or more were retained in the final model. Results were retested in non-small cell lung cancer (NSCLC) and small cell lung cancer (SCLC) patients.

To examine the association between dietary habits and tumor histology, binary logistic regression models were employed.

## Results

### Patient characteristics

In total, the data of survival and alcohol drinking of 1052 patients were available. Of these patients, 951 had NSCLC, and 101 had SCLC. Median follow-up was 9.1 months (range 0–58.8 months). During follow-up, 869 patients died. Of the 1052 patients, 391 (37.1%) were classified as never drinkers, 289 (27.5%) as occasional drinkers, and 372 (35.4%) as frequent drinkers. In terms of preserved or fried food consumption, 768 (73.0%) patients were classified as occasional consumers, and the remaining 284 (27.0%) were classified as frequent consumers. In terms of fresh fruit or vegetable consumption, 667 (63.4%) patients were classified as occasional consumers, and 385 (36.6%) were classified as frequent consumers. In terms of meat consumption, 941 (89.4%) patients were classified as occasional consumers, and 111 (10.6%) were classified as frequent consumers.

Baseline demographic and clinical characteristics grouped by the consumption level of alcohol and preserved or fried food are shown in Table 1. Patients who were 70 years of age and older, had an education level below college, and current smokers were more likely to be frequent consumers of alcohol and preserved or fried food. Current smokers were more likely to be frequent meat consumers. Occasional and frequent consumers of preserved or fried food had similar BMI levels.

**Table 1:** Baseline demographic and clinical characteristics of 1052 Chinese men with lung cancer, grouped by level of alcohol consumption and consumption of preserved or fried food

Characteristic^a^	Alcohol^b^			Preserved or fried food^c^	
	Never (*n* = 391)	Occasional (*n* = 289)	Frequent (*n* = 372)	Occasional (*n* = 768)	Frequent (*n* = 284)
*Age group (years)*					
<50	33 (8.4)	28 (9.7)	18 (4.8)	65 (8.5)	20 (7.0)
50–59	62 (15.9)	58 (20.1)	62 (16.7)	142 (18.5)	39 (13.7)
60–69	144 (36.8)	97 (33.6)	127 (34.2)	272 (35.5)	92 (32.4)
≥70	152 (38.9)	106 (36.7)	164 (44.2)^^^	288 (37.5)	133 (46.8)^^^
*District of residence*					
New Territories	65 (17.3)	49 (17.3)	50 (13.9)	126 (16.9)	38 (13.9)
Kwai Tsing	30 (8.0)	30 (10.6)	34 (9.5)	67 (9.0)	27 (9.9)
Wong Tai Sin	68 (18.1)	58 (20.5)	67 (18.7)	147 (19.7)	46 (16.8)
Kowloon City	41 (10.9)	50 (17.7)	36 (10.0)	95 (12.8)	32 (11.7)
Yau Tsim Moog	27 (7.2)	20 (7.1)	40 (11.1)	65 (8.7)	22 (8.1)
Sham Shui Po	59 (15.7)	26 (9.2)	58 (16.2)	98 (13.2)	45 (16.5)
Others	86 (22.9)	50 (17.7)	74 (20.6)^^^	147 (19.7)	63 (23.1)
*Education level*					
Primary school	102 (26.2)	50 (17.3)	110 (29.6)	179 (23.4)	84 (29.6)
Middle school	162 (41.5)	134 (46.4)	168 (45.3)	326 (42.6)	136 (47.9)
College or above	126 (32.3)	105 (36.3)	93 (25.1)^^^	261 (34.1)	64 (22.5)^^^
*Marital status*					
Married	62 (15.9)	44 (15.2)	68 (18.3)	130 (16.9)	45 (15.8)
Others	329 (84.1)	245 (84.8)	304 (81.7)	638 (83.1)	239 (84.2)
*Family income (Hong Kong dollars/month)*					
<4000	228 (58.6)	161 (55.7)	252 (67.7)	457 (59.7)	184 (64.8)
≥4000	100 (25.7)	88 (30.4)	79 (21.2)	198 (25.8)	69 (24.3)
Unknown	61 (15.7)	40 (13.8)	41 (11.1)^^^	111 (14.5)	31 (10.9)
*Smoking status* ^d^					
Never	60 (15.4)	34 (11.8)	21 (5.6)	103 (13.4)	13 (4.6)
Former	99 (25.4)	90 (31.1)	109 (29.3)	208 (27.1)	87 (30.6)
Current	231 (59.2)	165 (57.1)	242 (65.1)^^^	456 (59.5)	184 (64.8)^^^
*Cancer history in first*-*degree relatives*					
No	262 (67.2)	189 (65.4)	232 (62.5)	476 (62.1)	206 (72.5)
Yes	68 (17.4)	65 (22.5)	80 (21.6)	161 (21.1)	48 (16.9)
Not sure	60 (15.4)	35 (12.1)	59 (15.9)	129 (16.8)	30 (10.6)^^^
*Charlson comorbidity index*					
0–2	384 (98.2)	287 (99.3)	364 (97.8)	753 (98.0)	282 (99.3)
3–6	7 (1.8)	2 (0.7)	8 (2.2)	15 (2.0)	2 (0.7)
*Stage at diagnosis*					
I	52 (13.3)	30 (10.4)	36 (9.7)	90 (11.7)	28 (9.9)
II	11 (2.8)	17 (5.9)	20 (5.4)	34 (4.4)	16 (5.6)
III	105 (26.9)	92 (31.8)	129 (34.7)	243 (31.6)	80 (28.2)
IV	118 (30.2)	79 (27.3)	94 (25.3)	219 (28.5)	72 (25.3)
Unknown	105 (26.9)	71 (24.6)	93 (25.0)	182 (23.7)	88 (31.0)
*Treatment*					
Surgery	26 (6.6)	20 (6.9)	37 (9.9)	57 (7.4)	20 (7.0)
Chemotherapy	52 (13.3)	40 (13.8)	38 (10.2)	94 (12.2)	34 (12.0)
Radiotherapy	79 (20.2)	49 (17.0)	76 (20.4)	140 (18.2)	62 (21.8)
Others	8 (2.0)	7 (2.4)	14 (3.8)	18 (2.3)	11 (3.9)
No treatment	122 (31.2)	95 (32.9)	106 (28.5)	233 (30.3)	88 (31.0)
Combination	104 (26.6)	78 (27.0)	101 (27.2)	226 (29.4)	69 (24.3)
*BMI* ^e^ *(mean* *±* *SD)*	21.2 ± 3.3	21.3 ± 3.1	21.1 ± 3.3	21.4 ± 3.2	20.7 ± 3.2*

*BMI* body mass index, *SD* standard deviation

^^^ *P* < 0.05 in the Chi Square test or Fisher’s exact test

* *P* < 0.05 in *t* test

^a^ Number of cases with missing data: 34 for district of residence; 2 for education level, family income, and cancer in first-degree relatives; 1 for age group and smoking status

^b^ Occasional consumer: 1–3 days/week; frequent consumer: ≥4 days/week

^c^ Occasional consumer: <1 serving/day; frequent consumer: ≥1 serving/day

^d^ Never smoker: smoking <20 packs of cigarettes in a lifetime or ≤1 cigarette per day for 1 year; former smoker: quit smoking ≥2 years ago; current smoker: still smoking or quit <2 years ago

^e^ Except this one, other values are presented as number of cases followed by percentage in parentheses

### Dietary habits and lung cancer prognosis

Confounding factors retained in the final model were district of residence, age at diagnosis, cancer history in first-degree relatives, BMI, cancer stage at diagnosis, smoking status, smoking pack-years, and treatment type. Education level and family income were also retained in the final model because they were likely to affect the association between alcohol consumption and overall survival.

**Fig. 1:**
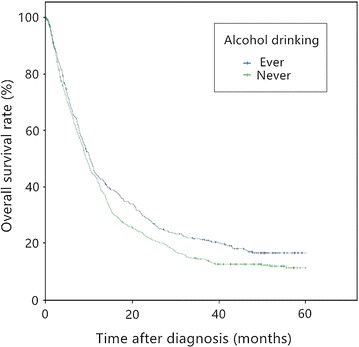
Kaplan–Meier survival curves for Chinese men with lung cancer, grouped by level of alcohol consumption. Patients who ever regularly consumed alcohol before being diagnosed with lung cancer had a better prognosis than those who never consumed alcohol

Lung cancer prognosis in relation to alcohol consumption and dietary habits is shown in Table 2. Compared with never drinkers, drinkers had a 17% lower risk of lung cancer death (HR: 0.83, 95% CI 0.70–0.98). The observed favorable lung cancer prognosis in alcohol drinkers was restricted to occasional drinkers (HR: 0.82, 95% CI 0.68–0.97). Figure 1 illustrates the survival curve regarding alcohol consumption habits. Compared with occasional consumers of preserved or fried food, frequent consumers had a higher risk of lung cancer death (HR: 1.20, 95% CI 1.00–1.42). Consumption of fresh fruits or vegetables and meat was not statistically associated with lung cancer death (Table 2).

**Table 2:** Risk of lung cancer death in Chinese men in relation to the levels of alcohol consumption and dietary habits

Component	No. of cases	Unadjusted HR	95% CI	Adjusted HR^c^	95% CI
*Alcohol* ^a^					
Never	391	1.00		1.00	
Ever	661	0.85	0.74–0.98	0.83	0.70–0.98
Occasional	289	0.83	0.71–0.97	0.82	0.68–0.97
Frequent	372	0.96	0.81–1.13	0.91	0.73–1.14
*Preserved/fried food* ^b^					
Occasional	768	1.00		1.00	
Frequent	284	1.06	0.91–1.23	1.20	1.00–1.42
*Fruits/vegetables* ^b^					
Occasional	667	1.00		1.00	
Frequent	385	1.00	0.87–1.15	0.86	0.72–1.02
*Meat* ^b^					
Occasional	941	1.00		1.00	
Frequent	111	1.11	0.89–1.38	1.27	0.98–1.64

*HR* hazard ratio, *CI* confidence interval

^a^ Ever consumer: consumed alcoholic beverages in the year before cancer diagnosis; this group includes occasional and frequent consumer groups. Occasional consumer: 1–3 days/week; frequent consumer: ≥4 days/week

^b^ Occasional consumer: <1 serving/day; frequent consumer: ≥1 serving/day

^c^ Adjusted for district of residence, age at diagnosis, body mass index, cancer history in first-degree relatives, education level, family income, stage at diagnosis, smoking status, smoking pack-years, and treatment

### Subgroup analysis according to histologic types

Hazard ratios were retested in NSCLC and SCLC cases (Table 3). For patients with NSCLC, the prognosis of occasional drinkers was better than that of never drinkers (HR: 0.74, 95% CI 0.62–0.90). However, this survival advantage became non-significant in frequent drinkers (HR: 0.84, 95% CI 0.70–1.02). Frequent consumption of preserved or fried food had an adverse effect on the prognosis of NSCLC patients (HR: 1.21, 95% CI 1.00–1.45). Because the number of SCLC cases was small, all results in SCLC patients were not statistically significant.

**Table 3:** Adjusted risk of lung cancer death in relation to levels of alcohol consumption and dietary habits in Chinese men according to histologic subtypes

Component	NSCLC			SCLC		
	No. of cases	Adjusted HR^c^	95% CI	No. of cases	Adjusted HR^c^	95% CI
*Alcohol* ^a^						
Never	349	1.00		42	1.00	
Ever	602	0.83	0.70–0.98	59	0.52	0.28–0.96
Occasional	265	0.74	0.62–0.90	24	0.63	0.31–1.29
Frequent	337	0.84	0.70–1.02	35	1.37	0.66–2.79
*Preserved/fried food* ^b^						
Occasional	700	1.00		68	1.00	
Frequent	251	1.21	1.00–1.45	33	1.12	0.62–2.05
*Fruits/vegetables* ^b^						
Occasional	630	1.00		37	1.00	
Frequent	321	0.93	0.78–1.11	64	0.74	0.41–1.34
*Meat* ^b^						
Occasional	848	1.00		93	1.00	
Frequent	103	1.19	0.92–1.58	8	0.73	0.31–1.73

*OR* odds ratio, *CI* confidence interval, *NSCLC* non-small cell lung cancer, *SCLC* small cell lung cancer

^a^ Ever consumer: consumed alcoholic beverages in the year before cancer diagnosis; this group includes occasional and frequent consumer groups. Occasional consumer: 1–3 days/week; frequent consumer: ≥4 days/week

^b^ Occasional consumer: <1 serving/day; frequent consumer: ≥1 serving/day

^c^ Adjusted for district of residence, age at diagnosis, body mass index, cancer history in first-degree relatives, education level, family income, stage at diagnosis, smoking status, smoking pack-years, and treatment

### Alcohol consumption, dietary habits, and tumor histology

The two confounding factors retained in the final model were age at diagnosis and smoking status. Table 4 shows the associations between alcohol drinking and dietary habits with lung cancer histology. Compared with SCLC patients, NSCLC patients were less likely to frequently consumed fruits or vegetables (odds ratio [OR]: 0.62, 95% CI 0.41–0.95) and preserved or fried food (OR: 0.50, 95% CI 0.33–0.76).

**Table 4:** Associations of alcohol consumption and dietary habits with lung cancer histology in 1052 patients

Component	No. of cases (%)		Adjusted OR^c^	95% CI
	NSCLC	SCLC		
*Alcohol* ^a^				
Never	349 (33.2)	42 (4.0)	1.00	
Ever	602 (57.2)	59 (5.5)	1.48	0.97–2.24
*Preserved/fried food* ^b^				
Occasional (<1 serving/day)	700 (66.5)	68 (6.5)	1.00	
Frequent (≥1 serving/day)	251 (23.9)	33 (3.1)	0.50	0.33–0.76
*Fruits/vegetables* ^b^				
Occasional (<1 serving/day)	630 (59.9)	37 (3.5)	1.00	
Frequent (≥1 serving/day)	321 (30.5)	64 (6.1)	0.62	0.41–0.95
*Meat* ^b^				
Occasional (<1 serving/day)	848 (80.6)	93 (8.8)	1.00	
Frequent (≥1 serving/day)	103 (9.8)	8 (0.8)	0.93	0.46–1.88

*OR* odds ratio, *CI* confidence interval, *NSCLC* non-small cell lung cancer, *SCLC* small cell lung cancer

^a^ Ever consumer: consumed alcoholic beverages in the year before cancer diagnosis

^b^ Occasional consumer: <1 serving/day; frequent consumer: ≥1 serving/day

^c^ Adjusted for age at diagnosis and smoking status, using SCLC as the reference

## Discussion

In this study, we found that Chinese men with lung cancer who ever drank alcohol had a better prognosis than those who never drank alcohol (HR: 0.84, 95% CI 0.72–0.98); however, the observed favorable prognosis in alcohol drinkers was restricted to occasional drinkers (HR: 0.80, 95% CI 0.67–0.96). Furthermore, men who frequently consumed preserved or fried food had a higher risk of lung cancer death than occasional consumers (HR: 1.21, 95% CI 1.02–1.43).

A similar U-shaped dose-responsive pattern between alcohol consumption and survival was found in some studies on breast cancer [15–17]. A meta-analysis indicated that moderate consumption of wine may have a chemopreventive effect on lung cancer, whereas consumption of beer may increase lung cancer risk [18]. Some in vitro studies suggested that polyphenols in wine can inhibit cancer cell proliferation and thus prolong survival [19, 20]. Nevertheless, beer accounts for the majority of alcohol consumption in Hong Kong [21]; the benefit of wine polyphenols is unlikely to explain the observed survival advantage in drinkers.

Until now, there has been no evidence to suggest that the consumption of preserved or fried food affects lung cancer prognosis. Recently, animal studies confirmed that dietary acrylamide (a substance generated when food is fried) is mutagenic in mouse lungs [22]. Nitrite, a potential carcinogen in preserved food, might facilitate the process of lung cancer development; a high serum nitrite level might have a negative effect on the survival of lung cancer patients [23–25]. Consistent epidemiologic evidence has shown that the habit of consuming fresh fruits and vegetables has a preventive effect on lung cancer [6]. Regarding the relationship between fruit and vegetable consumption and lung cancer prognosis, two small-scale studies indicated that frequent consumption of fruits or vegetables might be beneficial. Noticeably, the findings of one of these studies were not statistically significant [26]; the other one observed benefits only in women [27]. The benefit of fruit or vegetable consumption in lung cancer prognosis was supported by two more recent small trials, but they did not employ controls [28, 29]. Consumption of meat, especially red meat and preserved meat, has long been hypothesized to be carcinogenic; however, whether the frequent consumption of meat is related to lung cancer risk remains controversial [30]. High level of meat consumption is associated with the high intake of fat, endogenous carcinogens from heme, and exogenous carcinogens generated in the process of cooking and preservation [31]. These substances are presumed to function in the pathways of tumor progression. However, no relevant epidemiologic evidence has been presented.

A few studies have sought to determine how alcohol affects cancer prognosis. Of several mechanisms proposed, the immune system seems to be decisive. Alcohol affects the immune system in two opposite ways. When the alcohol dose is low, the immune system is stimulated to inhibit tumor growth; when the dose is high enough, alcohol leads to immune inhibition and promotes tumor progression [32]. This evidence may partly explain the U-shaped dose-responsive pattern observed in this study and in previous breast cancer studies [15]. Low-to-moderate alcohol consumption (practiced by occasional drinkers in this study) may initiate the first phase of the immune response, which restrains tumor growth and yields better survival, whereas heavy drinking (practiced by frequent drinkers in this study) may exceed the threshold and trigger the second phase, which promotes tumor progression.

Another possible explanation for the favorable lung cancer prognosis in alcohol drinkers is the variation of genes involved in the metabolism of alcohol and anti-cancer drugs. The frequency of alcohol consumption is related to the status of cytochrome P450 and glutathione S-transferase enzymes [17]. People with deficiencies in metabolism enzymes may experience unfavorable physical responses to alcohol, meaning that they will be unlikely to be regular drinkers. The primary alcohol metabolism cytochrome P450 is CYP2E1 [33]; CYP2E1 is also essential in the metabolism of the anti-cancer drugs cisplatin and etoposide [34, 35], which are frequently used in lung cancer chemotherapy. For SCLC, cisplatin plus etoposide is the prioritized first-line regimen [36]. Thus, lung cancer patients whose cytochrome P450 metabolism function is weak may have a low tolerance for chemotherapy; consequently, they may have a poor response to treatment and a shorter survival time. Moreover, before getting cancer, they are very unlikely to be regular drinkers.

Although there are controversies, the association between alcohol consumption and lung cancer risk has been well discussed. Earlier studies also focused on the effect of dietary habits on the incidence of lung cancer. Regarding lung cancer prognosis, it is well known that prognosis is associated with tumor characteristics (e.g., histologic subtypes and gene mutations), stage, and treatment. However, the prognostic values of alcohol consumption and dietary habits in lung cancer have either never been studied or studied only rarely. Thus, existing evidence shows that alcohol consumption and dietary habits are more related to the incidence of lung cancer than to prognosis. However, because lifestyle habits are modifiable, it is possible that the prognosis of lung cancer patients can be further improved by adopting more healthful habits. Future larger studies that quantify the lifetime consumption of alcohol and foods are needed to verify our findings.

Our study did have several limitations. Selection bias is a concern, but it should not be a major issue. The distributions of age and histologic subtype of our patients were similar to those reported by the Hong Kong Cancer Registry. Because all patients in this study were Chinese men, one should be cautious about generalizing the results to women and to other races. Confounding from cigarette smoking and other related factors could also be a concern because drinking is generally associated with factors like smoking, age, education level, income, and social level. To minimize the confounding effect, we tried to adjust as many related factors as possible into the “base” regression model. In the final model, some social factors were not retained because the removal of them could not change the estimate by 10% or more in the backward stepwise survival analysis. However, the confounding effect may still be a concern because it is impossible to adjust for every potential confounder. Misclassification of alcohol and dietary consumption levels may be an issue; but this misclassification, if it does exist, should be regarded as a non-differential one, which may lead to an attenuated association. More detailed information on alcohol drinking and dietary habits, with quantity estimation and categorization, was not available because we thought that, in our pilot study, information on lifetime food and alcohol consumption from older men, especially, would not be accurate. This limitation prevented us from conducting further analyses.

In conclusion, we found that Chinese men who consumed alcohol occasionally prior to the diagnosis of lung cancer had a better prognosis than those who never drank alcohol. However, this survival benefit was not observed in frequent drinkers. In this population, frequent consumers of preserved or fried food had a higher risk of lung cancer death than occasional consumers. We suggest that future studies be conducted to confirm our findings.
